# Multimodal Image Alignment via Linear Mapping between Feature Modalities

**DOI:** 10.1155/2017/8625951

**Published:** 2017-07-06

**Authors:** Yanyun Jiang, Yuanjie Zheng, Sujuan Hou, Yuchou Chang, James Gee

**Affiliations:** ^1^School of Information Science and Engineering, Key Lab of Intelligent Computing & Information Security in Universities of Shandong, Institute of Life Sciences, Shandong Provincial Key Laboratory for Distributed Computer Software Novel Technology and Key Lab of Intelligent Information Processing, Shandong Normal University, Jinan, Shandong 250014, China; ^2^Computer Science and Engineering Technology Department, University of Houston-Downtown, Houston, TX 77002, USA; ^3^Perelman School of Medicine, University of Pennsylvania, Philadelphia, PA 19104, USA

## Abstract

We propose a novel landmark matching based method for aligning multimodal images, which is accomplished uniquely by resolving a linear mapping between different feature modalities. This linear mapping results in a new measurement on similarity of images captured from different modalities. In addition, our method simultaneously solves this linear mapping and the landmark correspondences by minimizing a convex quadratic function. Our method can estimate complex image relationship between different modalities and nonlinear nonrigid spatial transformations even in the presence of heavy noise, as shown in our experiments carried out by using a variety of image modalities.

## 1. Introduction

Multimodal/multispectral images acquired from multiple modalities or different spectral bands of the same subject or organ are of great importance for medical diagnosis and computer-aided surgery, benefiting from the complementary information captured by sensors of different modalities/spectra (e.g., magnetic resonance imaging and computed tomography or the multispectral imaging) [1–3]. They are also being more and more widely used in other fields, such as computer vision and computational photography, accomplished via different imaging modalities (e.g., RGB and near infrared) or under various imaging conditions (e.g., flash and no flash, depth, and color images) [4].

Image alignment resolves spatial correspondences between images and plays a fundamentally important role in practical application of multimodal images. There currently exist various techniques [4–9] for multimodal image alignment, which can be basically categorized into feature-based and patch-based methods. The feature-based methods detect sparse salient points and extract features to describe their local photometric/geometric pattern [10, 11]. Different from alignment of generic images, multimodal image alignment requires the features together with their similarity measurement to be able to deal with image variations caused by the modality difference [6]. The patch-based methods measure the similarity between local patches by computing their mutual information [12], cross correlation [4, 6, 13], or their combination [14].

**Figure 1:**
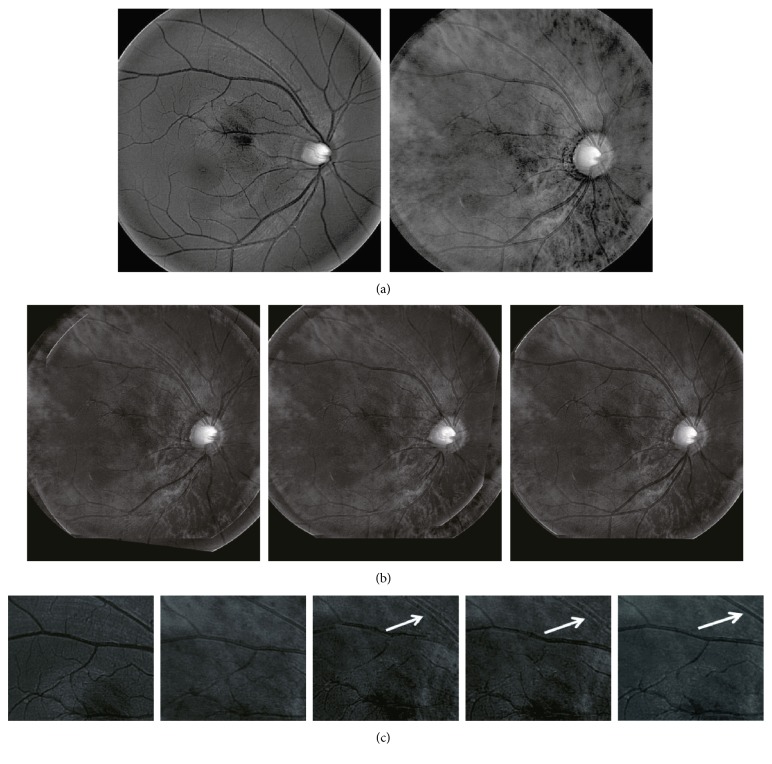
From left to right: (a) a pair of MSI images captured from the same retina but at different spectra; (b) overlaid results generated after alignments by the algorithms based on mutual information [19], robust measurement [4], and our linear mapping, respectively; (c) a small rectangular patch chosen at a similar position from the images from left to right at (a) and (b), respectively. The white arrow in the rectangular patches points to an area bearing obvious differences in vessel alignment between different algorithms.

Disregarding the promising results reported in existing papers, multimodal image alignment still remains a challenge mainly due to the complex and unknown relationship between image modalities (as shown by the left two images in Figure 1(c)). The common information between multimodal images is needed for defining image features. However, it is not always trivial to recognize, model, or learn this information in practice due to outliers, large displacement, and the complex relationship [4]. Moreover, the predefined image features can work well only when the corresponding measurement of the feature similarity fits these features, which is not always an easy task in practice. Finally, the definition of image feature and similarity is independent from the computation of spatial correspondences in most of the existing works for multimodal image alignment, which may lead to suboptimal solutions.

In this paper, we propose a new landmark matching based multimodal image alignment method which uniquely builds an implicit linear mapping of features extracted for describing each landmark in one image to the ones of the corresponding landmark in the other image taken in a different modality/condition. It runs as resolving the linear mapping and the landmark correspondences simultaneously by minimizing squared differences between features. Our method bears several advantages over the state-of-the-art techniques. First, the resolved linear mapping enables our method to gain an effective similarity measure by adaptively discovering common information between images, even based only on common image features for describing image local properties at each landmark and the *L*_2_ norm for measuring feature differences. Second, simultaneous optimization of the linear mapping and landmark correspondences results in an optimal solution, benefiting from their mutual interactions involved in the optimization process. Third, we formulate the problem as an integer quadratic programming and resolve it with an efficient conditional gradient algorithm.

## 2. Problem Definition

Suppose we have a pair of images, denoted by *I* and *J*, which are taken under different modalities. From each of them, we extract a set of landmark points, represented by *P*_*I*_ = {1,…, *m*} for *I* and *P*_*J*_ = {1,…, *n*} for *J*, respectively. We aim to align *I* and *J* by searching correspondences between *P*_*I*_ and *P*_*J*_.

## 3. Linear Mapping

Great challenges in corresponding the landmarks in *P*_*I*_ and the ones in *P*_*J*_ arise from the complex relationship between *I* and *J* in the sense of not only the brightness value at each landmark but also the local photometric/geometric pattern at the vicinity of each landmark. In order to tackle this hard problem, we first extract a set of features denoted by a vector *θ*_*i*_ ∈ ℝ^*k*^ for each landmark *i* in *P*_*I*_ and a set of features *ϕ*_*j*_ ∈ ℝ^*l*^ for landmark *j* in *P*_*J*_, respectively. By stacking the features of all landmarks together, we have Θ = [*θ*_1_,…, *θ*_*m*_] ∈ ℝ^*k*×*m*^ and Φ = [*ϕ*_1_,…, *ϕ*_*n*_] ∈ ℝ^*l*×*n*^. Then, if landmark *i* in *P*_*I*_ corresponds to landmark *j* in *P*_*J*_, we solve a projection matrix *T* ∈ ℝ^*k*×*l*^ such that *θ*_*i*_ = T*ϕ*_*j*_. In other words, we assume that there is a linear mapping from *ϕ*_*j*_ to *θ*_*i*_. At the same time, we assume that all pairs of corresponding landmarks follow the same linear mapping, which can be written as

(1) $$\begin{array}{c} \Theta E=T\Phi , \end{array}$$

where *E* denotes a correspondence matrix. *E* is a binary matrix, that is, *E* ∈ *𝔹*^*m*×*n*^, for which rows correspond to the landmarks of *P*_*I*_ and columns are associated with *P*_*J*_. Elements of *E* take a value of 1 when the related landmarks correspond and 0 if otherwise.

## 4. Objection Function

We resolve the correspondence matrix *E* together with the projection matrix *T* of the linear mapping by optimizing the following objective function with the Frank-Wolfe algorithm [15]:

(2) $$\begin{array}{c} \underset{E}{\min}\;\underset{T}{\min}{\left\|\Theta E-T\Phi \right\|}_{2}^{2}+\lambda {\left\|T\right\|}_{2}^{2} \end{array}$$

where the right term enforces an *L*_2_ regularizer on *T* and λ is an adjusting parameter. When *E*where the right term enforces an *L*_2_ regularizer on *T* and λ is an adjusting parameter. When *E* is fixed, (2) becomes a ridge regression problem [16] with respect to *T* and generates a solution

(3) $$\begin{array}{c} T=\Theta E\Phi^{\prime }{\left(\Phi \Phi^{\prime }+\lambda I\right)}^{-1}, \end{array}$$

where **I** is an identity matrix. By combining (2) and (3), we have

(4) $$\begin{array}{c} O_{m}=\underset{E}{\min}\;Tr\;\left(\Theta {EZE}^{\prime }\Theta^{\prime }\right), \end{array}$$

where Tr() means the computation of trace and *Z* is written as

(5) $$\begin{array}{c} Z=I-\Phi^{\prime }{\left(\Phi \Phi^{\prime }+\lambda I\right)}^{-1}\Phi . \end{array}$$

## 5. Enforcing Priors

In order to avoid degenerated solutions which are characterized as being obviously different from a reasonable solution in practice, we enforce two constraints on *E* in (4) based on our prior knowledge about the landmark matching problem. The first one aims to minimize the number of landmarks in *P*_*I*_/*P*_*J*_ associated with each landmark in *P*_*J*_/*P*_*I*_; that is, we do not hope many landmarks in one image are assigned to a landmark in the other image. This constraint can be expressed as

(6) $$\begin{array}{c} O_{c1}=\underset{E}{\min}\;\left({\left\|E1-\mu \right\|}_{2}^{2}+{\left\|E^{\prime }1-\mu \right\|}_{2}^{2}\right), \end{array}$$

where 1 is an all-ones vector and *μ* is a parameter to be set empirically. In (6), *μ* controls the number of points. The second constraint is introduced to prevent two landmark points from being associated if they are too distant to be true. It can be written as

(7) $$\begin{array}{c} O_{c2}=\underset{E}{\min}\;\left(Tr\;\left(X_{I}E-X_{J}\right)+Tr\;\left(X_{J}E^{\prime }-X_{I}\right)+\left(Y_{I}E-Y_{J}\right)+Tr\;\left(Y_{J}E^{\prime }-Y_{I}\right)\right), \end{array}$$

where *X*_*I*_ and *X*_*J*_ are matrices created by repeating copies of the horizontal vector composed by *X* coordinates of landmarks in *P*_*I*_ and *P*_*J*_, respectively, and *Y*_*I*_ and *Y*_*J*_ are built in a similar way by using *Y* coordinates instead.

## 6. Optimization

Combining (4), (6), and (7), our landmark matching problem is formulated as a minimization of the following objective function:

(8) $$\begin{array}{c} O=O_{m}+\lambda_{1}O_{c1}+\lambda_{2}O_{c2}. \end{array}$$

Equation (8) is a positive semidefinite quadratic function and its minimization is NP hard. We optimize (8) by using a similar technique to [15], which consists of a relaxation of the binary matrix *E* to be continuous and the optimization to be over the convex hull of *E* (via the Frank-Wolfe algorithm [15]), and a procedure of rounding the resulted continuous solution of *E* by minimizing the Euclidean distance between the binary *E* and the continuous *E* with a linear programming optimization. As shown in [15], the Frank-Wolfe algorithm can find the global optimization of the correspondence matrix *E* in (2), and therefore, we can simply initialize it randomly.

## 7. Landmark Detection and Features

In our algorithm, landmark points are specified as the key locations of SIFT [17] due to its prestigious advantage of being stable. The features for describing each landmark point are the gradient orientation matrices (GOM) [18].

## 8. Experimental Results

We implemented our algorithm in MATLAB® and its processing time for a 2500 × 2300 image is less than 2 minutes on a 2.39 GHz Core i7 computer. In our experiments, we empirically set *λ* = 0.6, *λ*_1_ = 0.8, *λ*_2_ = 0.1, and *μ* = 1.5. We employ a coarse-to-fine strategy based on an image pyramid created by using 3 scales with a downsampling rate of 0.8.

In order to validate our algorithm, we collected a dataset which consists of a pair of aerial and orthophoto images copied from MATLAB, 6 pairs of flash/no-flash indoor images taken by using a Canon camera, 6 pairs of RGB/depth images captured by Microsoft Kinect, and 10 pairs of multispectral imaging (MSI) ocular images acquired by using an Annidis RHA™ instrument (Annidis Health Systems Corp., Ottawa, Canada). Every pair of images comes from the same scene/object, for example, MSI images of each pair share the same retina. In our experiments, we converted all RGB images to gray.

**Figure 2:**
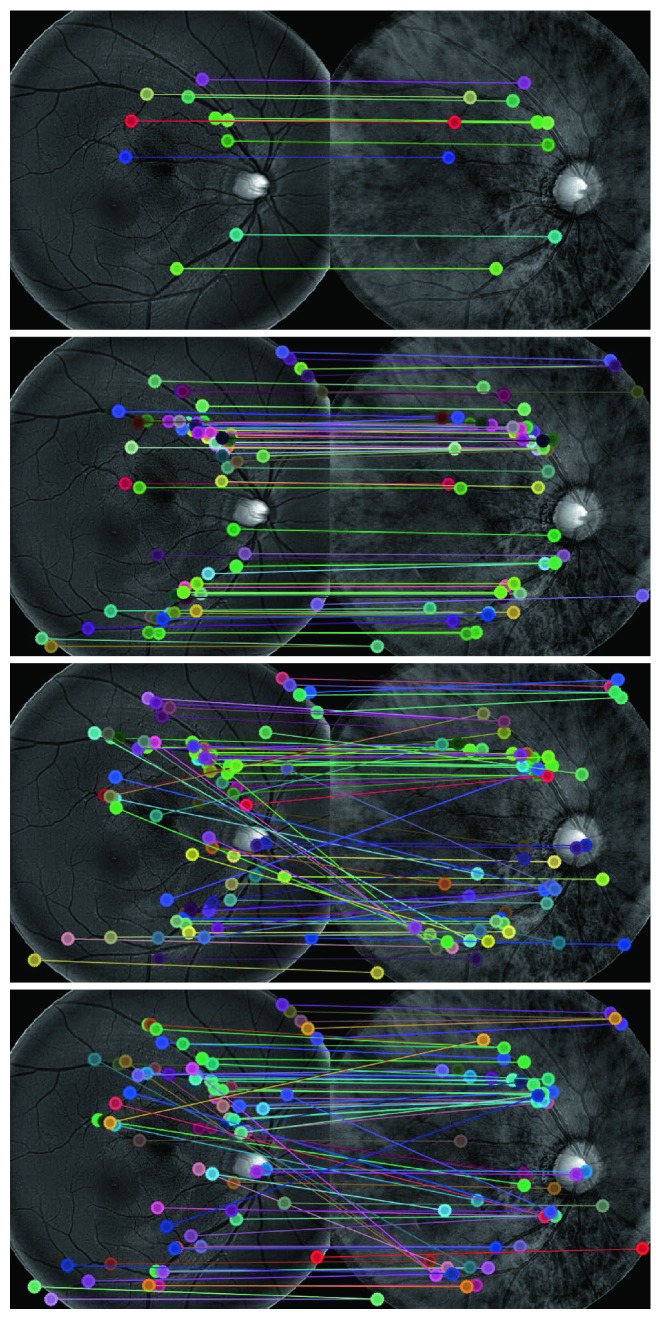
Matches of points in the pair of MSI images in Figure 1. Top to bottom: the 10 manually marked point pairs and the 70 best-matched point pairs from the 437 SIFT points detected from each image, by our linear mapping, the mutual information [19] and the robust measurement [4], respectively.

**Figure 3:**
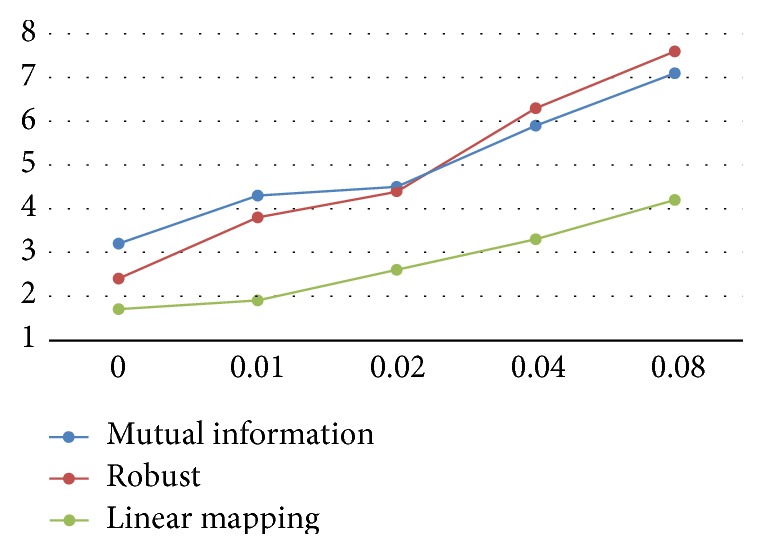
Mean distance (in pixels as shown by the *Y*-axis) between manually marked points and results of the algorithms based on mutual information [19], the robust measurement [4] and our linear mapping, respectively. Gaussian noise with zero mean and variances of 0, 0.01, 0.02, 0.04, and 0.08 are added in the images, respectively, as shown by the *X*-axis.

We compared our linear mapping based method with the classic mutual information based approach [19] and the recently proposed robust measurement based technique [4] both qualitatively and quantitatively. We first ran the three algorithms on our dataset and visually compared their performances by both overlaying the transformed image to the other image and showing the connection of matched points. Observed misalignment in the overlaid image or the matched-point connections means an inferior performance of the matching algorithm. In our experiments, we found that our algorithm outperforms the other two methods for 19 pairs (an exampling pair of MSI images are shown in Figures 1 and 2) and produces comparable results for the left 4 pairs. Then, we added into all images Gaussian noise with zero mean and variances of 0, 0.01, 0.02, 0.04, and 0.08, respectively. For each pair of images, a trained rater manually marked 10 points which are easy to recognize in both images (as shown in Figure 2). We treated the 230 manually set point pairs as the ground truth and computed the quantitative errors (as shown in Figure 3) of the three methods that we are evaluating. Specifically, we estimated the 12-parameter transformation model for the retina [20, 21] for MSI images and an affine model for other images, used it to transform manually set points in one image to the other image and then computed the spatial distance between the transformed point and the corresponding manually set point.

As shown by the results in Figures 1–3, we have at least three findings. First, our algorithm performs better than the two representative state-of-the-art techniques, as shown by its fewer vessel misalignments in the overlaid images especially in the area to which the white arrow points in the rectangular patches of Figure 1 and the smaller quantitative errors in Figure 3. Second, our algorithm can automatically discover the complex relationship (as shown by the left two images in Figure 1(c)) between images taken from different modalities and therefore results in better accuracies. Third, the linear mapping demonstrates better robustness to image noise, and the simultaneous optimization of linear mapping and landmark correspondences shows an extraordinary ability to estimate nonlinear nonrigid transformations (e.g., the retina in Figure 1).

## 9. Conclusion

We have presented a novel landmark matching based multimodal image alignment technique. It is distinguished from existing image alignment techniques by at least two of its unique characteristics. First, it automatically discovers the latent complex relationship between different feature modalities. Second, it simultaneously solves for the linear mapping and landmark correspondences based on a minimization of a convex quadratic function. Our future works would include extensions of our algorithm to other features (e.g., learned features [22]) and for describing landmark points, different features for different modalities, and a supervised alignment scheme.
